# Reliability and validity of the Turkish version of the Barthel Index-dyspnea in patients with pulmonary hypertension

**DOI:** 10.55730/1300-0144.6213

**Published:** 2026-02-15

**Authors:** Pınar MERÇ, Rengin DEMİR, Melih ZEREN, Habibe DURDU, Ümit Yaşar SİNAN, Mehmet Serdar KÜÇÜKOĞLU

**Affiliations:** 1 Department of Cardiopulmonary Physiotherapy and Rehabilitation, Gülhane Faculty of Physiotherapy and Rehabilitation, University of Health Sciences, Ankara, Turkiye; 2 Department of Cardiology, Cardiology Institute, İstanbul University-Cerrahpaşa, İstanbul, Turkiye; 3 Department of Physiotherapy and Rehabilitation, Faculty of Health Sciences, İzmir Bakırçay University, İzmir, Turkiye; 4 Department of Therapy and Rehabilitation, Vocational School of Health Services, Giresun University, Giresun, Turkiye

**Keywords:** Pulmonary hypertension, activities of daily living, dyspnea, validity, reliability

## Abstract

**Background/aim:**

The Barthel Index-dyspnea (BI-d) is a respiratory disease-specific tool developed on the basis of the Barthel Index (BI), which provides a simple and quick way to measure the degree of dyspnea in activities of daily living (ADL). Dyspnea is one of the most common symptoms of pulmonary hypertension (PH). The objective of this study was to explore the reliability and validity of the Turkish version of the BI-d in patients with PH.

**Materials and methods:**

A total of 73 PH patients were included in the study. Criterion validity was explored using the modified Medical Research Council (mMRC) dyspnea scale, 6-min walk test (6MWT), and London Chest Activities of Daily Living (LCADL) scale, while divergent validity was examined through correlations between the BI-d and BI.

**Results:**

The BI-d demonstrated strong correlations with the LCADL (r = 0.801) and mMRC (r = 0.775), and a moderately negative correlation with the 6MWD (r = −0.510), supporting criterion-related validity. A weak negative correlation with the BI (r = −0.289) supported divergent validity. The BI-d scores differed significantly across the mMRC grades and PH functional classes, confirming known-group validity. Reliability analyses showed good internal consistency (Cronbach’s α = 0.85) and excellent test–retest reliability (intraclass correlation coefficient = 0.94).

**Conclusion:**

The Turkish version of the BI-d demonstrates high reliability and validity in PH. As a tool that integrates respiratory burden with activity-related functional performance, it provides a practical assessment of dyspnea-related impairment during ADL.

## 1. Introduction

Pulmonary hypertension (PH) is a physiopathological condition characterized by an average pulmonary artery pressure (mPAP) greater than 20 mmHg at rest [1]. The most common symptom is progressive dyspnea, occurring in approximately 85% of patients, while exertional dyspnea is also reported in nearly all patients [2]. Symptoms such as fatigue, weakness, angina, edema, palpitations, and syncope have also been reported [3,4].

The effects of the disease on employment, household income, activities of daily living (ADL), and social isolation have been reported in the literature. Most PH patients report that their physical function is severely affected by symptoms including dyspnea, fatigue and lack of energy, limiting their ability to complete even the most basic daily tasks such as housework and shopping [5,6]. Therefore, it is essential to measure and understand dyspnea, as it is one of the major symptoms in PH. Evaluation of dyspnea therefore requires a multifaceted approach that combines clinical assessment with specific measurement tools to capture its subjective impact, with the aim of improving patient care and guiding future research [7].

The Barthel Index (BI) is one of the most common scales used to evaluate motor and functional disabilities of patients during ADL. However, it does not take dyspnea into account and cannot be used as an appropriate measurement tool in respiratory patients [8]. Although there are scales used for the assessment of dyspnea during ADL like London Chest Activities of Daily Living (LCADL) scale [9], none of these tools offer the opportunity to simultaneously assess both motor and respiratory disability during ADL. The purpose of this study was to translate the Barthel Index-dyspnea (BI-d) into Turkish and evaluate its validity and reliability in patients with PH.

## 2. Materials and methods

### 2.1. Participants

Seventy-three patients with PH who applied to the Cardiology Department of İstanbul University-Cerrahpaşa Cardiology Institute were included in the study. Patients aged 18 to 70 years who were diagnosed with PH by a specialist physician according to the 2022 PH Treatment and Diagnosis Guidelines of the European Heart and Respiratory Society [1], and who had received regular medication treatment during the previous 3 months were included in the study. Patients with diagnosed orthopedic or neurological diseases or who had communication difficulties during the evaluations were excluded.

Patients willing to participate in the study signed informed consent forms. The İstanbul University Cerrahpaşa Non-Interventional Clinical Research Ethics Committee reviewed and approved the research protocol (Approval number: 2024/180).

### 2.2. Measures

Physical and sociodemographic data such as age, body mass index (BMI), smoking status, PH type, mPAP, and World Health Organization functional class (WHO-FC) were recorded for all the patients. Criterion-related validity of BI-d was evaluated against three different tools: the LCADL, which evaluates the impact of dyspnea on daily activities; the modified Medical Research Council (mMRC) dyspnea scale, which assesses the severity of breathlessness during physical activity; and the 6-min walk test (6MWT), which measures functional capacity. In addition, the BI was used to assess divergent validity of the BI-d.

#### 2.2.1. mMRC

The mMRC is a patient-reported outcome measure used to assess the degree of breathlessness during daily activities. Scores vary between 0 and 4, where 0 = shortness of breath only during vigorous exercise; 1 = shortness of breath when hurrying or walking uphill; 2 = walks slower than peers or stops to rest at their own pace; 3 = stops after ~100 m on flat ground or after a few minutes; and 4 = experiences shortness of breath when getting dressed and is unable to leave home [10].

#### 2.2.2. LCADL

The LCADL is a simple and standardized assessment tool consisting of 15 items developed to evaluate dyspnea resulting from ADL. Each item is ranked on a scale of 0 to 5; the higher the score, the more difficulty the person has performing ADL [9].

#### 2.2.3. 6MWT

The 6MWT is used to evaluate functional exercise capacity, in accordance with the criteria of the American Thoracic Society. During the test, patients are instructed to walk as far as possible at their own pace for 6 min along a straight 33-m corridor. The distance covered by the patients during the 6 min is recorded in meters (6MWD) [11].

#### 2.2.4. BI

The BI is a 10-item tool developed to assess physical performance during ADL. The BI score, which can be performed simply and quickly, ranges from 0 to 100. A score of 100 indicates that the person is fully independent in activities [12].

#### 2.2.5. BI-d

The BI-d was developed by Vitacca et al. [8] to assess dyspnea during ADL based on the BI items. Findings suggest that the BI-d is a reliable and valid measurement tool for individuals with chronic respiratory disorders. The BI-d includes 10 items assessing dyspnea during ADL. These items are derived from the BI and consist of items such as care, showering, feeding, restroom use, stair use, dressing, defecation, urination, movement, wheelchair use, and transfers. Each item is assessed on a Likert-type scale of five points (0–4), in which 0 = no dyspnea; 1 = mild dyspnea that does not prevent or slow down ADL; 2 = moderate dyspnea that may slow down ADL; 3 = severe dyspnea that may significantly slow down ADL; 4 = extremely severe dyspnea that prevents or reduces this activity. The total BI-d score ranges from 0 (no shortness of breath) to 100 (maximum level of shortness of breath). For test–retest reliability, the questionnaire was repeated twice at an interval of 7–10 days.

### 2.3. Translation, cultural adaptation, reliability, and validity procedure

For the translation process, the guidelines of Beaton et al. [13] for cross-cultural adaptation were used. The original English version of the BI-d was sent to two bilingual individuals (one physiotherapist, one from an unrelated field) for translation into Turkish (version 1). Version 1 was reviewed and consolidated by the research team and two translators (version 2). Then, two native English speakers, who were not familiar with the subject, translated version 2 back into English (version 3). The research team and a committee of experts evaluated all these versions and established the final Turkish version (version 4). A pretest was conducted with a sample of 20 individuals during an outpatient visit and revealed no difficulty in understanding the content of the statements.

### 2.4. Statistical analysis and sample size

IBM SPSS Statistics for Windows 20.0 (IBM Corp., Armonk, NY) was used for data analysis. Normality of the data was assessed using the Kolmogorov–Smirnov test. The construct validity of the BI-d questionnaire was examined through criterion-related, divergent, and known-groups validity. Criterion-related and divergent validity were evaluated by analyzing correlations between BI-d scores and the mMRC, LCADL, 6MWD and BI using Pearson’s correlation coefficients. Known-groups validity was examined by comparing BI-d scores between patient groups defined by mMRC and WHO-FCs using one-way analysis of variance (ANOVA). Reliability was examined through internal consistency (Cronbach’s α) and test–retest reliability using the intraclass correlation coefficient (ICC). Cronbach’s α was considered acceptable at ≥ 0.70 (good at ≥ 0.80, excellent at ≥ 0.90), and test–retest reliability was considered poor for ICC < 0.50, moderate for 0.50–0.74, good for 0.75–0.89, and excellent for ≥ 0.90. Moreover, test–retest scores of the BI-d were compared for any significant variations. p < 0.05 was considered statistically significant. Considering general recommendations for scale development, at least 5 participants per item were targeted for the study [14]. Accordingly, a minimum sample size of 50 participants was calculated; however, all participants who met the inclusion criteria during the study period were consecutively recruited.

## 3. Results

A total of 73 participants were included. The mean age was 49.01 years, and 80.8% were female. Table 1 shows the sociodemographic and clinical characteristics of participants. In this cohort, 45.2% of the patients were classified as WHO-FC II. According to clinical classification, most participants (80.8%) had group I PH, followed by 11.0% with group IV chronic thromboembolic PH. The results of the 6MWT and patient-reported outcomes are presented in Table 2.

### 3.1. Validity

**Figure 1:**
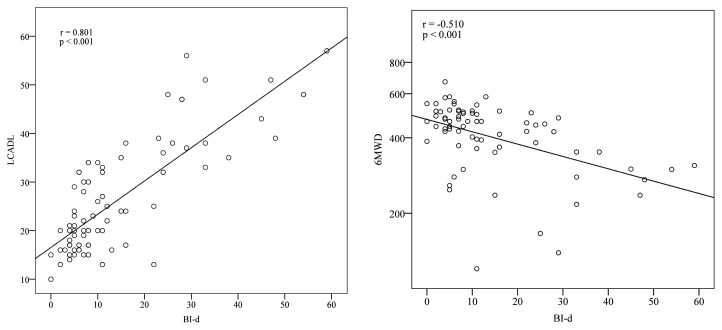
Correlation of the BI-d with the LCADL and 6MWD.

When the data were analyzed (Figure 1), a strong positive correlation was found between the BI-d and LCADL (r = 0.801, p < 0.001); however, the relationship with the 6MWD was negative and moderate (r = −0.510, p < 0.001). The strong relationship between the BI-d and mMRC (r = 0.775, p < 0.001) occurred in the expected direction, providing additional evidence for criterion validity (Table 3). Supporting the discriminant validity of the BI-d, a weak, negative, and significant relationship (r = −0.289, p = 0.013) was found between the BI and BI-d (Table 3).

**Figure 2:**
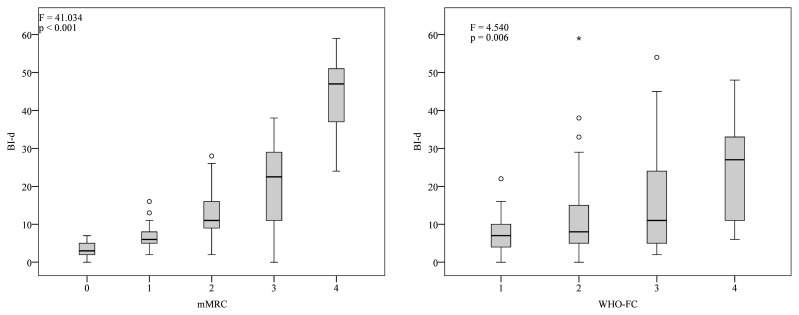
Box plot of the BI-d distribution according to the mMRC and WHO-FC.

The BI-d scores varied significantly according to the mMRC grades (F = 41.034, p < 0.001) and PH functional classes (F = 4.540, p = 0.006) (Figure 2), supporting the known-group validity.

### 3.2. Reliability

Internal consistency was good, with a Cronbach’s α of 0.85. Test–retest reliability was excellent, as shown by an ICC of 0.94 (95% CI: 0.91–0.96, p < 0.001). Comparison of the test–retest scores of the BI-d yielded no significant difference (p = 0.790).

## 4. Discussion

The Turkish version of the BI-d was determined to be valid as the result of its significant correlations with the mMRC, LCADL and 6MWD in patients with PH. In addition, the BI-d scores differed across the mMRC grades and WHO-FCs, confirming the scale’s ability to distinguish between patients with different levels of dyspnea severity and functional limitations. The BI-d was also highly reliable, as supported by Cronbach’s α, ICC, and test–retest score comparisons. During the translation process, no modifications were necessary to adapt the scale to Turkish or in a sociocultural context.

Various measures and tools have been designed to assess shortness of breath, a symptom commonly seen in patients with chronic respiratory diseases that limits their quality of life. Frequently used and considered the gold standard in dyspnea assessment, the mMRC is a 0–4-point scale that assesses the impact of dyspnea on mobility limitations but does not provide an activity-based assessment [7,8]. The LCADL is a tool that has been validated and reliability tested in Turkish and aims to assess dyspnea based on ADL [9]. However, to the best of our knowledge, no validation and reliability studies have been reported for the LCADL in PH patients. Unlike the LCADL, the BI-d does not include any subscales, which may allow for faster assessment.

The BI, a widely used measure for assessing motor independence in ADL in chronic patients, does not evaluate dyspnea, one of the primary symptoms limiting ADL in patients with chronic respiratory disease. To address this limitation, the BI-d was developed by Vitacca et al. [8] as an adaptation of the original BI. A validity study conducted on a similar clinical group in Japan demonstrated that the scale was valid and reliable; but importantly, no cultural adaptation was performed in that study either [15].

The relationship between dyspnea and functional capacity has been demonstrated in various cardiopulmonary clinical groups. Albarrati et al. [16] demonstrated that dyspnea is more closely related to exercise tolerance than to cardiac function parameters in chronic heart failure patients. In a study on patients with long COVID, dyspnea was found to be unrelated to pulmonary function parameters, whereas it showed a significant association with exercise capacity [17]. Greater dyspnea has been associated with reduced functional capacity, as reflected by a lower 6MWD in PH [18]. In the current study, one of the reference measures used for evaluating the validity of the BI-d was the 6MWD. The 6MWT is the most frequently used method for evaluating functional exercise capacity in PH clinics and plays an important role in the assessment and management of PH [1,19]. In the original study, the validity of the BI-d was also assessed using the 6MWD. In that study, where the mean 6MWD was 279 ± 143, a negative, moderately strong, and statistically significant relationship was observed between the BI-d and 6MWD. In the Japanese version, a moderate negative correlation was found between the BI-d and 6MWD. In the Turkish version, a similar negative correlation of moderate to strong was observed, indicating that patients with lower functional capacity experienced higher perceived dyspnea during ADL. In patients with chronic dyspnea, the dyspnea level during ADL assessed by the LCADL showed a strong negative correlation with the 6MWD [20]. It was reported that the 6MWD in chronic obstructive pulmonary disease (COPD) patients showed a strong correlation with the mMRC and a moderate correlation with the total LCADL score [21]. Taken together, the findings of previous studies support the current findings of a significant association between the BI-d and 6MWD.

Herein, the mMRC was also used to evaluate the validity of the BI-d. A strong positive correlation was found between the Turkish BI-d and mMRC, and the BI-d scores differed significantly across the mMRC and WHO-FCs, with scores being higher in patients with greater symptom severity. These findings support the validity of the BI-d, particularly its known-groups validity, which—unlike previous studies—was evaluated for the WHO-FCs for the first time. These results are in line with earlier findings, such as those of Vitacca et al. [8], who reported a strong correlation between the MRC and BI-d, and the Japanese version of the BI-d [15] similarly showed a strong correlation with the mMRC. The mMRC is commonly used in the assessment of dyspnea in cardiopulmonary diseases, as it is a unidimensional, easy-to-use, and time-efficient tool for grading the impact of dyspnea on daily activities. Beyond PH, the relevance of the mMRC has also been reported in other diseases; in adults with asthma, the LCADL demonstrated a moderate correlation with quality of life and a weak to moderate correlation with the mMRC [22], while in COPD, although both the COPD Assessment Test and mMRC reflect ADL levels, the mMRC demonstrates a stronger correlation, independently predicts ADL, and is additionally associated with lung function [23]. Similarly, in the current study, the significant correlations of the BI-d with mMRC and its ability to discriminate between mMRC groups further support its validity.

In previous research, test–retest reliability was excellent in the original study (ICC = 0.99) and high in the Japanese adaptation (ICC = 0.76). In the current Turkish validation, test–retest analyses likewise supported strong temporal consistency; the ICC was high (ICC = 0.94), and the paired comparison between administrations showed no significant mean difference, consistent with the absence of learning or fatigue effects. The test–retest reliability of the Turkish version of the LCADL was reported to be high in patients with COPD (ICC = 0.95) [9]. A study on COPD patients reported that the mMRC had a test–retest reliability coefficient of 0.82 (ICC) [24]. Considering the literature, the reliability of the Turkish version of the BI-d is noteworthy. In line with the original version, the Cronbach’s α in our sample also indicated high internal consistency, supporting the suitability of the BI-d for repeated use in similar clinical contexts.

The current study had some limitations. The sample was predominantly composed of Group 1 PH patients. This spectrum imbalance limited external validity to the broader PH population, particularly Groups 2–5, where dyspnea mechanisms, comorbidity burdens (e.g., left heart disease, lung disease/hypoxemia), and treatment courses differ. Another limitation was that the responsiveness was not evaluated. Consequently, the ability of the Turkish version of the BI-d to detect clinically meaningful changes over time remains to be established. Further research is needed to assess responsiveness using distribution-based metrics alongside anchor-based strategies and to define minimal important change thresholds specific to PH. The Turkish version of the BI-d is a valid, reliable, and internally consistent tool for assessing dyspnea in ADL. It also offers a quick and easy method for use in clinical settings. As a modification of the BI, we believe that it will contribute to the assessment of motor autonomy in patients with PH. Future studies should examine the BI-d in specific PH groups and in pulmonary rehabilitation.

## Figures and Tables

**Table 1:** Baseline characteristics of the participants.

Variables	Mean ± SD, n (%)

Age, year	49.01 ± 12.80

BMI, kg/m^2^	27.46 ± 5.98

Sex	
Female/male	59 (80.8%), 14 (19.2%)

Smoking status	
Yes/no	6 (8.2%), 67 (91.8%)

WHO-FC	
I	17 (23.3%)
II	33 (45.2%)
III	13 (17.8%)
IV	10 (13.7%)

Pulmonary arterial pressure (mmHg)	53 ± 26.43

Etiology of PH	
Group 1 (PAH)	59 (80.8%)
Group 2 (PH left heart disease)	2 (2.7%)
Group 3 (PH lung disease)	2 (2.7%)
Group 4 (CTEPH)	8 (11.0%)
Group 5 (Unclear and/or multifactorial mechanisms)	2 (2.7%)

BMI: body mass index; SD: standard deviation; WHO-FC: World Health Organization functional class; PAH: pulmonary arterial hypertension; PH: pulmonary hypertension; CTEPH: chronic thromboembolic PH.

**Table 2:** Assessment of functional capacity, dyspnea, and ADL.

Variables	Mean ± SD
6MWD (m)	421.70 ± 111.59
LCADL	26.25 ± 11.54
mMRC	1.79 ± 1.18
Barthel Index	99.04 ± 2.85
BI-d (first assessment)	14.21 ± 13.52
BI-d (second assessment)	14.36 ± 14.26

6 MWD: 6-min walk distance, LCADL: London Chest Activity of Daily Living Scale; mMRC: Modified Medical Research Council.

**Table 3:** Correlation of BI-d with the BI and mMRC.

Parameter	BI-d	
	r	p-value
**BI**	−0.289	0.013*
**mMRC**	0.775	0.000**

* p < 0.05,

** p < 0.01.
